# Comparing apples to apples: an environmental criminology analysis of the effects of heat and rain on violent crimes in Boston

**DOI:** 10.1057/s41599-018-0188-3

**Published:** 2018-11-20

**Authors:** Alice J. Sommer, Mihye Lee, Marie-Abèle C. Bind

**Affiliations:** 1 Faculty of Arts and Sciences, Department of Statistics, Harvard University, Cambridge, MA, USA.; 2 Graduate School of Public Health, St. Luke’s Int’l University, Tokyo, Japan.

## Abstract

Weather characteristics have been suggested by many social scientists to influence criminality. A recent study suggested that climate change may *cause* a substantial increase in criminal activities during the twenty-first century. The additional number of crimes *due* to climate have been ethoroughly discussed the first draft of the paper. Allstimated by associational models, which are not optimal to quantify *causal* impacts of weather conditions on criminality. Using the Rubin Causal Model and crime data reported daily between 2012 and 2017, this study examines whether changes in heat index, a proxy for apparent temperature, and rainfall occurrence, influence the number of violent crimes in Boston. On average, more crimes are reported on temperate days compared to extremely cold days, and on dry days compared to rainy days. However, no significant differences in the number of crimes between extremely hot days versus less warm days could be observed. The results suggest that weather forecasts could be integrated into crime prevention programs in Boston. The weather–crime relationship should be taken into account when assessing the economic, sociological, or medical impact of climate change. Researchers and policy makers interested in the effects of environmental exposures or policy interventions on crime should consider data analyses conducted with causal inference approaches.

## Introduction

For approximately four decades, criminologists have been interested in the weather–crime relationship (Bell and Baron, 1975; Cohen and Felson, 1979; DeFronzo, 1984; Harries et al., 1984; Anderson et al., 1995; Cohn and Rotton, 1997; Mares, 2013a). Two main theories have been suggested to explain the association between heat and human behavior. First, the *routine activities theory* argues that pleasant weather increases outdoor activity, thus exposing more people to offenders and leaving homes unprotected until extreme heat induces people to seek shelter indoors (Cohen and Felson, 1979). Consequently, Cohn and Rotton (1997; 2000a) expect the relationship between temperature and offenders’ social contacts to be an inverted-U shaped function. In contrast, the *heat-aggression theory* argues that changes in temperature affect crimes by increasing irritability and anger. To test the second argument and examine the shape of the dose–response, Bell and Baron (1975; 1976) conducted human experiments and observed that, by exposing research subjects to artificially uncomfortable conditions, aggression increases along with temperature until temperature crosses the threshold from warm to unbearable, after which subjects display lower levels of aggression. Other authors argued for a positive linear relationship between temperature and aggressive behavior (Anderson et al., 1995; Bushman et al., 2005). Several empirical analyses with different study designs and different data aggregation levels (e.g., hourly, daily, weekly, annual data) provided evidence for the existence of a weather-crime relationship. We present a summary of studies that investigated the effect of heat on crimes with results supporting either of the theories in Table 1. In addition to the impact of temperature, criminologists have been interested in the impact of rainfall. A positive relationship between rain and robbery was reported (DeFronzo, 1984), although other studies did not find evidence for a relationship between precipitation and the frequency of homicide or rape (Feldman and Jarmon, 1979; Perry and Simpson, 1978; DeFronzo, 1984). According to the routine activities theorists, rainfall could either prevent property and violent crimes, or increase assaultive behavior, by increasing the likelihood of people staying inside. From a heat-aggression point of view, a cooling-effect could occur from temperatures being reduced by precipitation (Baron and Bell, 1976). Criminologists have also extensively inspected the relationship between neighborhood conditions and crimes, they generally agree on the existence of a geographic component in levels of violence and criminal behavior (Sampson, 1997). The geographic differences in the weather–crime relationship are well explained by Harries et al. (1984), and more recently by Mares (2013b), who suggest higher violence rates in disadvantaged neighborhoods when climatic conditions are warmer.

Weather conditions leading to criminal activities can affect the mental and physical health of the offender and the victim. For this reason, violent crimes are considered an important public health issue. Preventing crimes may involve not only the police and the criminal justice system, but also environmental scientists, epidemiologists, and criminologists. Over the past two decades, an increasing number of studies have associated climate with human conflicts, criminal behavior, and violence (Rotton and Cohn, 2003; Agnew, 2011; Gamble and Hess, 2012; Hsiang et al., 2013; Mares, 2013a; Ranson, 2014; Mares and Moffett, 2016; Levy et al., 2017; Tiihonen et al., 2018). Recently, Ranson (2014) predicted that 22,000 murders, 1.2 million aggravated assaults, and 2.3 million simple assaults will occur in the United States by the end of the twenty-first century, *because of* climate change and the resulting change in temperature and rainfall patterns. The precise estimation of the magnitude of weather effects (e.g., temperature anomalies) on violence is important for criminologists and researchers in fields related to climate change and public health (Satcher, 1995). Most studies estimate the relationship between weather variables and crimes with associational models (e.g., linear, multilevel, and time series regressions). *Controlling* or *adjusting* for confounding variables, by including them in a regression model is not optimal for addressing causality in observational studies, especially when the covariate distributions of the exposed and control groups are substantially far apart (Rubin, 2008). If the covariate distributions do not overlap in the exposed and control groups, researchers are often implicitly making strong assumptions (e.g., inappropriate linear extrapolations) that can lead to biases. Cochran and Rubin (1973); Heckman et al. (1998); and Rubin (2001) have shown that regression can estimate biased causal effects when the true relationship between the background covariates or the outcome is unknown, as well as when the means and variances of the background covariates are considerably different for the exposed and control groups.

In this manuscript, we depart from associational modeling to provide estimates of the weather–crime relationship, and use an alternative estimation and inference method that relies on the Rubin Causal Model (RCM) (Rubin, 1974). Following a multi-stage strategy, we carefully reconstruct the observational weather–crime data in such a way that mimics randomized experiments, in which we can then quantify daily effects of weather on crime (Bind and Rubin, 2017). We use the RCM coupled with a matched-sampling strategy that enables us to compare exposed and control days as if they had been randomized (Rosenbaum and Rubin, 1983). Designing the observational data carefully and independently of the outcome is required before performing the statistical analysis using the outcome. The objective is to *compare apples to apples*, thereby we mean, we construct groups of exposed and control days that resemble each other with respect to background covariates while blinding ourselves from the outcome of interest (i.e., daily crime rates). We focus on the weather–crime relationship in Boston because of its wide spectrum of weather conditions. In Boston, average Summer temperatures can rise up to 28 °C and it has been suggested that the number of days below 0 °C will decrease to 34 days per year by the end of the century (Research Advisory Group (BRAG), 2016). In this paper, we estimate the effects of heat index and precipitation on daily violent crime counts and provide the reader with easily interpretable interval estimates obtained with transparent assumptions. Our approach concentrates on the estimation of meteorological influences on crimes at a finite population level (i.e., the Boston area). Our goal is not to extrapolate our local findings to a more generalizable dose–response curve. However, we discuss and compare the plausibility and the effect size of our results for Boston with the weather–crime relationship in Los Angeles, a city with a smaller weather condition spectrum.

## Crime data analysis with the Rubin Causal Model

### Data description and analysis strategy.

Weather conditions from the Boston Logan airport monitoring station were obtained from the Climate Data Online provided by the National Oceanic and Atmospheric Administration (NOAA2018). The heat index (HI) is calculated from air temperature and dew point temperature, using the U.S. National Weather Service’s formula suggested by Anderson et al. (2013). We explore the heat–crime relationship in Boston by estimating the effect of changes in heat index, and not temperature alone to incorporate not only ambient temperature, but also ambient humidity. Both the routine activities and the heat-aggression perspectives rely on how humans feel and react to heat variations. Apparent temperature was developed to measure thermal comfort (Steadman, 1994). Because the heat index is used as a proxy for apparent temperature, we believe it provides a better quantification of human discomfort.

In this study, the crime data come from crime incident reports (between July 2012 and February 2017) collected by the Boston Police Department and made available on the City of Boston Data Portal (2018). Details on the location and time of day are given for all reported crimes. In the Fall and the Summer, the daily count of violent crimes and average HI are higher than during the Winter and the Spring on average (see Table S1). It might feel counterintuitive for Fall to have higher temperatures than Spring but it is worthwhile to note that Boston is located in New England, a region that is known for its *Indian Summer*. Figure S1 shows that the daily violent crime counts are the highest during the Summer months (i.e., June, July, August) on average. The approach we undertake to investigate the heat–crime relationship uses binary exposures (e.g., daily cold vs. temperate HI). Therefore, we decided to classify the days by heat index class: [−18; 0 °C], (0; 24 °C], (24; 35 °C], the *Negative*, *Mild*, and *High* heat index class, respectively (see Fig. S2(a)). For the the estimation of the effect of precipitation vs. no precipitation no data segmentation is necessary (see Fig. S2(b)).

Our strategy is to primarily focus on daily counts of violent crimes (i.e., aggravated assaults, simple assaults, crimes involving weapons, homicides, kidnapings, manslaughters, murders, escapes, runaways, truancies, and vandalism). Secondarily, we explore aggravated assault and larceny counts in order to analyze both a violent and a property offense in the study. In addition, we use the crime locations to discuss the potential neighborhood differences in the weather–crime relationship. Our choice of background covariates is based on the literature suggesting that the weather–crime relationship can be confounded by meteorological conditions, seasons, public holidays, large events, day of the month (crime reporting can be systematically biased on the first, middle, and last day of the month), and day of the week.

### Potential outcomes and notation.

The data segmentation described above leads to three *hypothetical randomized experiments* that help to understand the effects of daily changes in heat index: we reconstruct one experiment for each heat index class. A fourth hypothetical experiment is reconstructed for the analysis of the effect of rainfall where each day will either be assigned to precipitation or no precipitation. As illustrated in Fig. S2 and Table S2, we define *Z*_*i*_ as the heat index and *T*_*i*_ as the threshold of day *i, i* = 1, …, *N*. The exposure indicator *W*_*i*_ is equal to 1 if *Z*_*i*_ > *T*_*i*_ (*i* is exposed), and 0 otherwise (*i* is control). For the first three experiments, we choose the average heat index as the threshold. For the last experiment on rain occurrence, the threshold is by definition set at 0 mm. In all four hypothetical experiments we assume that each day *i* is randomly assigned to the exposed group (*W*_*i*_ = 1) or the control group (*W*_*i*_ = 0) with probability 1/2.

The hypothetical randomized experiments we reconstruct are completely-randomized experiments (see Table S3). We need to assure that the Stable Unit Treatment Value Assumption (SUTVA) holds in order to frame our analysis within the Rubin Causal Model (RCM) (Rubin, 1974). Therefore, we assume that the crime count of a certain day occurring after some exposure level is independent of the exposure received on other days. Following the RCM, each day has two potential outcomes: *Y*_*i*_(1), the number of crimes that occurred in day *i* if *W*_*i*_ = 1 and *Y*_*i*_(0) otherwise. The observed number of crimes that occurred in *i* is denoted by $Y_{i}^{\text{obs}}$ and it is equal to *Y*_*i*_(0) when *W*_*i*_ = 0, or equal to *Y*_*i*_(1) when *W*_*i*_ = 1. The days where $Y_{i}^{\text{obs}}=Y_{i}(0)$ is observed are referred to as *control days* and the ones where $Y_{i}^{\text{obs}}=Y_{i}(1)$ are referred to as *exposed days*.

In each hypothetical experiment we assess the effect of the exposure on crimes. The unit-level exposure effect (UEE) is defined as the difference between both potential outcomes of *i*:

(1) $${\text{UEE}}_{i}=Y_{i}(1)-Y_{i}(0)$$

In this study, we are interested in the mean difference in daily crime counts between the exposed days and the control days. Our estimand of interest is the average exposure effect (AEE):

(2) $$\text{AEE}=\frac{1}{N}\sum_{i=1}^{N}{\text{UEE}}_{i}=\frac{1}{N}\sum_{i=1}^{N}Y_{i}(1)-\frac{1}{N}\sum_{i=1}^{N}Y_{i}(0)=\bar{Y}(1)-\bar{Y}(0)$$

Within each experiment, we estimate the AEE of different exposure levels, which can be interpreted as the average number of daily violent crimes resulting from a high exposure level (e.g., temperate heat index) compared to a lower exposure level (e.g., cold heat index).

### Design stages.

In observational studies, meteorological exposures cannot be randomly assigned. In such situations, to address causality, Rubin (2008) suggests to undertake a design stage that attempts to reconstruct a hypothetical experiment without using the observed outcome. This strategy avoids p-value hacking and multiple testing, thereby limiting false discovery biases in the subsequent statistical analyses. The goal is to fulfill the ideal conditions of a randomized experiment, implying that the treatment assignment is unconfounded given background covariates. Unconfoundedness of the treatment assignment can often be approximately achieved using matched-sampling strategies (Rubin, 2006).

Four design stages are performed in this study, one for each hypothetical experiment, and we use a one-to-one matching strategy with caliper on the estimated propensity score to create groups of exposed and control days with similar distributions for background covariates. The aim of this strategy is to improve balance in covariate distributions, in other words, the covariate distributions of the exposed and control days are similar on average (Rosenbaum and Rubin, 1983), therefore making sure we *compare apples to apples*. The background covariates included in the four hypothetical experiments are the binary variables: *Fall, Winter, Spring, Summer, Friday, Weekend, FirstDayMonth* (i.e., first day of the month), *MidDayMonth* (i.e., fifteenthth day of the month), *LastDayMonth* (i.e., last day of the month), *Snow, Holidays*^1^, and *Events*^2^ as well as the continuous variable *Wind* (i.e., windspeed). In the first three experiments concentrating on the effects of different levels of heat indexes, *Rain* (i.e., rainfall occurrence) is an additional binary background covariate. In the last experiment, concentrating on the effect of rainfall, *HeatIndex* plays the role of a background covariate. The steps to follow through each design stage to reach covariate balance are: (1) to estimate the propensity score for each day *i*, (2) to assess the overlap in propensity score distributions, and (3) to proceed with the actual one-to-one propensity score matching. These steps can be repeated multiple times until a satisfying balance is obtained.

First, let **X**_**i**_ be the vector of background covariates for day *i*, the propensity score is defined as the probability of being an exposed day given the background covariates, *e*_*i*_ = *e*_*i*_(**X**_**i**_) = *P*(*W*_*i*_ = 1 | **X**_**i**_). We estimate the propensity score of each day *i* via a logistic regression that regresses the log odds of an exposed day on the background covariates:

(3) $$W_{i}\sim \text{Bernoulli}\left(e_{i}\right)\;\text{logit}\left(e_{i}\right)=f\left(X_{i}^{{\mathrm{HExp}}_{k}},\beta \right)\;\text{for}\;k=1,\ldots ,4$$

At every design stage, different specifications are possible for the model in Eq. (3) and we attempt to find an appropriate functional form for *f*(**X**_**i**_**, *β***) by comparing models with stepwise model selection by Akaike Information Criteria. The following propensity score models were selected for each hypothetical experiment:

$$X_{i}^{{\mathrm{HExp}}_{1}}=(1,\;{\text{Winter}}_{i},\;{\text{Fall}}_{i},\;{\text{MidDayMonth}}_{i},\;{\text{Wind}}_{i},\;{\text{Rain}}_{i})\;X_{i}^{{\mathrm{HExp}}_{2}}=(1,\;{\text{Winter}}_{i},\;{\text{Spring}}_{i},\;{\text{Summer}}_{i},\;{\text{LastDayMonth}}_{i},\;{\text{Rain}}_{i},\;{\text{Wind}}_{i}*{\text{Rain}}_{i},\;{\text{Snow}}_{i},\;{\text{Events}}_{i})\;X_{i}^{{\mathrm{HExp}}_{3}}=(1,\;{\text{MidDayMonth}}_{i},\;{\text{Wind}}_{i})\;X_{i}^{{\mathrm{HExp}}_{4}}=(1,\;{\text{Winter}}_{i},\;{\text{Spring}}_{i},\;{\text{Wind}}_{i},\;{\text{Snow}}_{i},\;{\text{HeatIndex}}_{i},\;{\text{Wind}}_{i}*{\text{HeatIndex}}_{i},\;{\text{Events}}_{i})$$

Summer days were not present in the Negative heat index days experiment, as well as Winter days and *Events* in the High heat index experiment.

Second, once a propensity score is calculated for each day *i*, the overlap in propensity score distributions for the control and exposed days is assessed. Control days with estimated propensity score outside the range of estimated propensity scores of the exposed days are discarded, and vice versa. Let us call these days: *outlying days*. The idea is to have the same range of data across exposure groups to avoid extrapolating beyond the support of the data during the analysis. Table S6 presents the overlap in propensity score distribution before and after deleting the outlying days for each experiment.

Last, we proceed with the one-to-one matching strategy with caliper. To assess whether the matching is successful and that the hypothesized experiment is plausible, we examine the balance in background covariate distribution for each exposure group. Diagnostic plots, such as *Love* plots (Ahmed et al., 2006), of the Fig. S3(a–d) show the difference in standardized means for the background covariates before and after matching. There is no evidence against covariate imabalance when the difference is close to zero, and more likely when the distributions overlap. Figures S4–7 present a precise comparison of the empirical distribution of the background covariates before and after matching. These figures show how, in each experiment, matching enables the exposed and control background covariate distributions to become closer. Insuring covariate balance is crucial for valid estimation of the causal effects of exposures and their confidence intervals.

### Analytic methods.

After a design stage is conducted for the four hypothetical experiments, we analyze each sample with balanced distributions of covariates for each experiment. We are interested in several crime outcomes: daily violent crime counts, as well as aggravated assault, and larceny counts. In this study, we conduct a Bayesian analysis within the four matched-samples resulting from our design stages. We assume independent Negative-Binomial distributions for the potential outcomes of crime counts.

The Bayesian analysis estimates posterior distributions of the AEE in the finite population. This analysis method was initially proposed by Rubin (1978) and is also described by Imbens and Rubin (2015). The Bayesian inference approach is appealing in our setting because it imputes the missing potential outcomes via simulation-based computational methods instead of estimating the slope coefficient of a regression model using the observed outcomes. For each experiment, we start with specifying a negative-binomial (NB) distribution for the potential outcomes conditional on background covariates. The independence assumption avoids contaminating the imputations of exposed days’ potential outcomes by imputed values of the control days, and vice versa. As we do not have prior knowledge on the values of the parameters, we impose the weakly informative priors suggested in the rstanarm R package on the parameters (Gabry and Goodrich). We assume a half-cauchy distributed prior for the scale parameter and normally (N) distributed priors for the intercept and slope parameters of the linear predictor. The selection of background covariates was done using leave-one-out cross-validation, the loo method for rstanarm. For the four hypothetical experiments of the primary results, the missing potential outcomes follow negative-binomial distributions parametrized with *η*_*i*_ = log(*μ*_*i*_) and a dispersion parameter *ϕ* We use the following Bayesian models for the imputation:

$$\;Y_{i}^{\text{obs}}\sim N\;B\left(\mu_{i},\phi \right)\;\;\mu_{i}=\text{exp}(\eta_{i})\;\text{and}\;\eta_{i}=\beta^{T}X_{i}^{{\mathrm{HExp}}_{k}}\;\text{for}\;k=1,\ldots ,4\;\;X_{i}^{{\mathrm{HExp}}_{1}}=(1,\;{\text{FirstDayMonth}}_{i},\;{\text{MidDayMonth}}_{i})\;\;X_{i}^{{\mathrm{HExp}}_{2}}=(1,\;{\text{Friday}}_{i},\;{\text{Weekend}}_{i},\;{\text{FirstDayMonth}}_{i},\;{\text{MidDayMonth}}_{i},\;{\text{Wind}}_{i}*{\text{Rain}}_{i})\;\;X_{i}^{{\mathrm{HExp}}_{3}}=(1,\;{\text{Weekend}}_{i})\;\;X_{i}^{HExp_{4}}=(1,\;{\text{Friday}}_{i},\;{\text{Weekend}}_{i},\;{\text{FirstDayMonth}}_{i},\;{\text{Snow}}_{i},\;{\text{HeatIndex}}_{i})\;\;\text{Priors}\;:\;\phi \sim \mathrm{Half}–\mathrm{Cauchy}(0,5)\;\beta_{0}\sim N(0,5)\;\beta_{1,\ldots ,5}\sim N(0,2.5)$$

The distributions of the parameters *ϕ*, *β*_0_ and $\beta_{1,\ldots ,5}$ are estimated twice: once for the control potential outcomes and once for the exposed potential outcomes. We estimated these distributions with 20,000 iterations and we burned 10,000, so we have 10,000 values for each parameter. After that, we can impute the missing potential outcomes among the control and exposed groups separately. For each replication (10,000), one value of $Y_{i}^{\text{miss}}$ are drawn for each day *i*, conditional on $Y_{i}^{obs}$, the observed covariates, and the parameters. For every replication, the AEE is calculated, which gives us a distribution of the AEE and a 95% posterior interval. See Supplementary Material Section F for the models of the exploratory results.

## Results

**Fig. 1:**
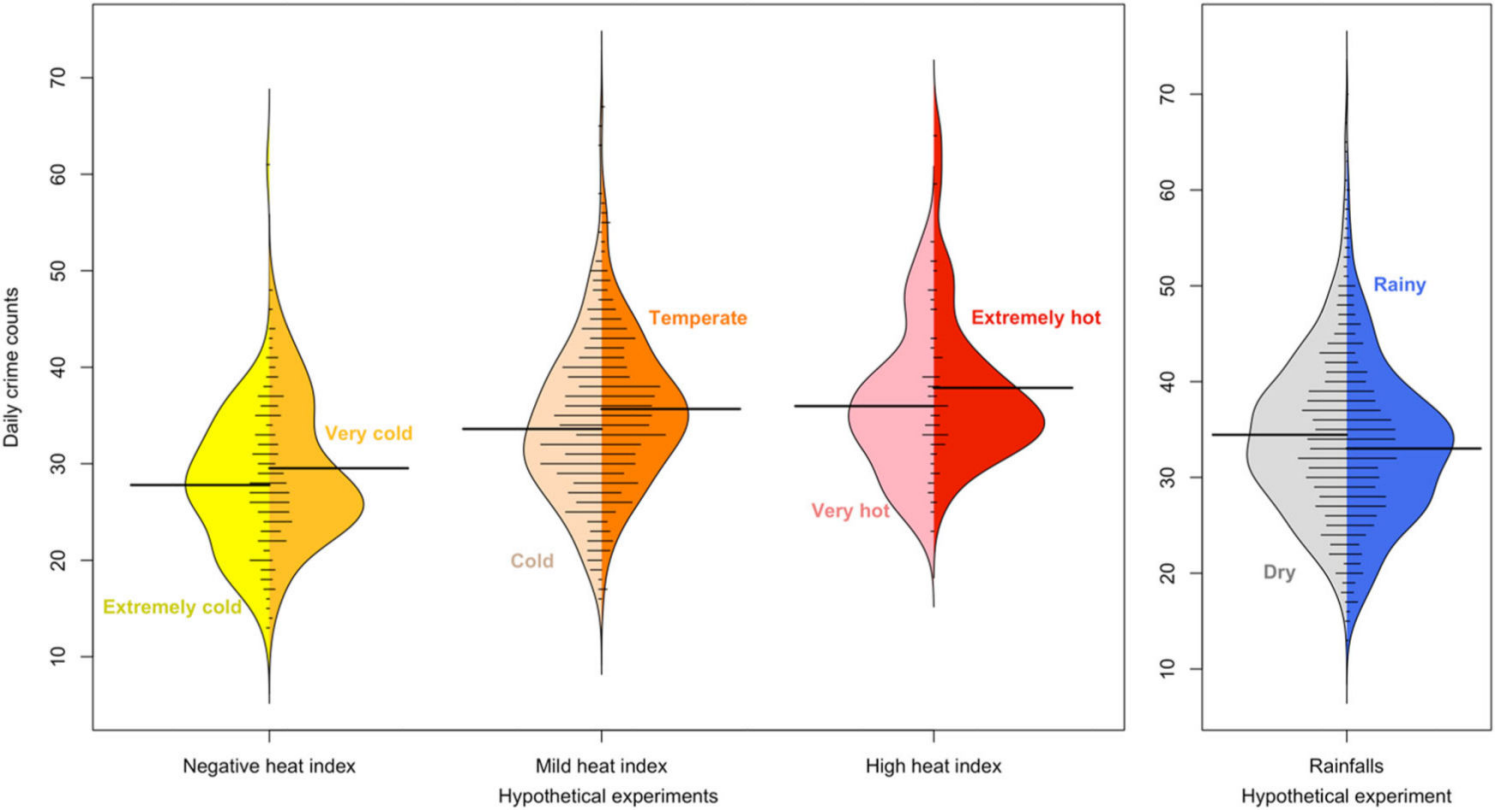
Daily violent crime count distributions for days with different exposure levels across the four hypothetical experiments, after propensity score matching. **a** Extremely cold (HI ≤−4 °C), very cold (−4 < HI < 0 °C), cold (0 < HI ≤ 12 °C), temperate (12 < HI < 24 °C), very hot (24 < HI ≤ 27 °C), and extremely hot (27 °C < HI) days. **b** Dry (PRCP < 0 mm) and rainy (PRCP ≥ 0 mm) days

**Fig. 2:**
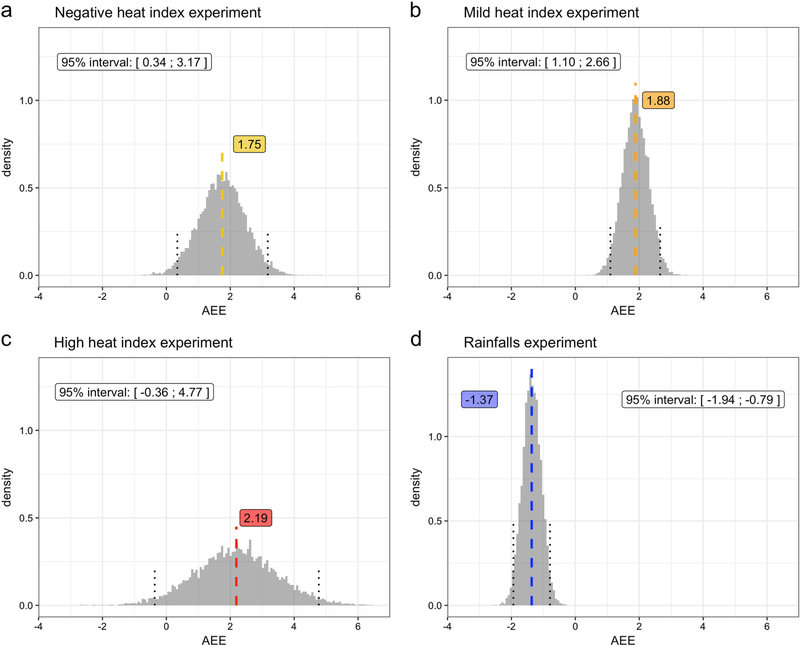
Primary results: Estimates of the average exposure effect of different exposure levels on daily violent crimes across the four hypothetical experiments after multiply imputing the missing potential outcomes 10,000 times

Design stages enable us to reconstruct four hypothetical randomized experiments before comparing the daily violent crime counts that occurred on days with different binary meteorological exposures (see Fig. 1). For each hypothetical experiment, we estimate the average exposure effect (AEE) on the total number of violent crimes and their corresponding 95% posterior interval (see Fig. 2 and Table S4). The more specific crimes presented in the exploratory analysis are aggravated assault and larceny (see Table S5). The spatial variation of the average daily violent crimes across zip-code areas is illustrated in the spatial description.

### Primary results.

In the Negative, Mild, and High heat index hypothetical experiments, the estimates of the AEE and their 95% posterior interval are: (1) 1.75 [0.34; 3.17], (2) 1.88 [1.10; 2.66] and (3) 2.19 [−0.36; 4.77], respectively. These results suggest that on average more violent crimes occur in Boston during very cold days (−4 < HI < 0 °C) compared to extremely cold days (HI ≤ −4 °C), and also during temperate days (12 < HI < 24 °C) compared to cold days (0 < HI ≤ 12 °C). However, we did not find enough statistical evidence to make conclusions about changes in daily violent crimes counts between extremely hot days (27 °C < HI) and hot days (24 < HI ≤ 27 °C). The hypothetical experiment focusing on the estimation of the AEE of the occurrence of rainfall suggests that compared to dry days, the average daily violent crimes count decreases by 1.37 (95% posterior interval: [−1.94; −0.79]) during rainy days. In Boston, rainy days have fewer crimes than dry days on average.

### Exploratory results.

The results of the Negative, Mild, and High heat index experiments suggest that, on average, 0.91 (95% interval = [0.45; 1.38]), 0.58 (95% interval = [0.30; 0.86]), and 1.31 (95% interval = [0.18; 2.41]) more aggravated assaults occur on very cold vs. extremely cold days, on temperate vs. cold days, and on extremely hot vs. very hot days, respectively. The estimated effect of rainfall on daily aggravated assault counts is −0.35 (95% interval = [−0.55; −0.14]). Larceny counts also vary between days that are extremely cold and days with temperate heat. In the High heat index and the Rainfalls experiments, there is no evidence of a trend in larceny counts when days are subject to high heat exposure or precipitation occurrence.

### Spatial description.

**Fig. 3:**
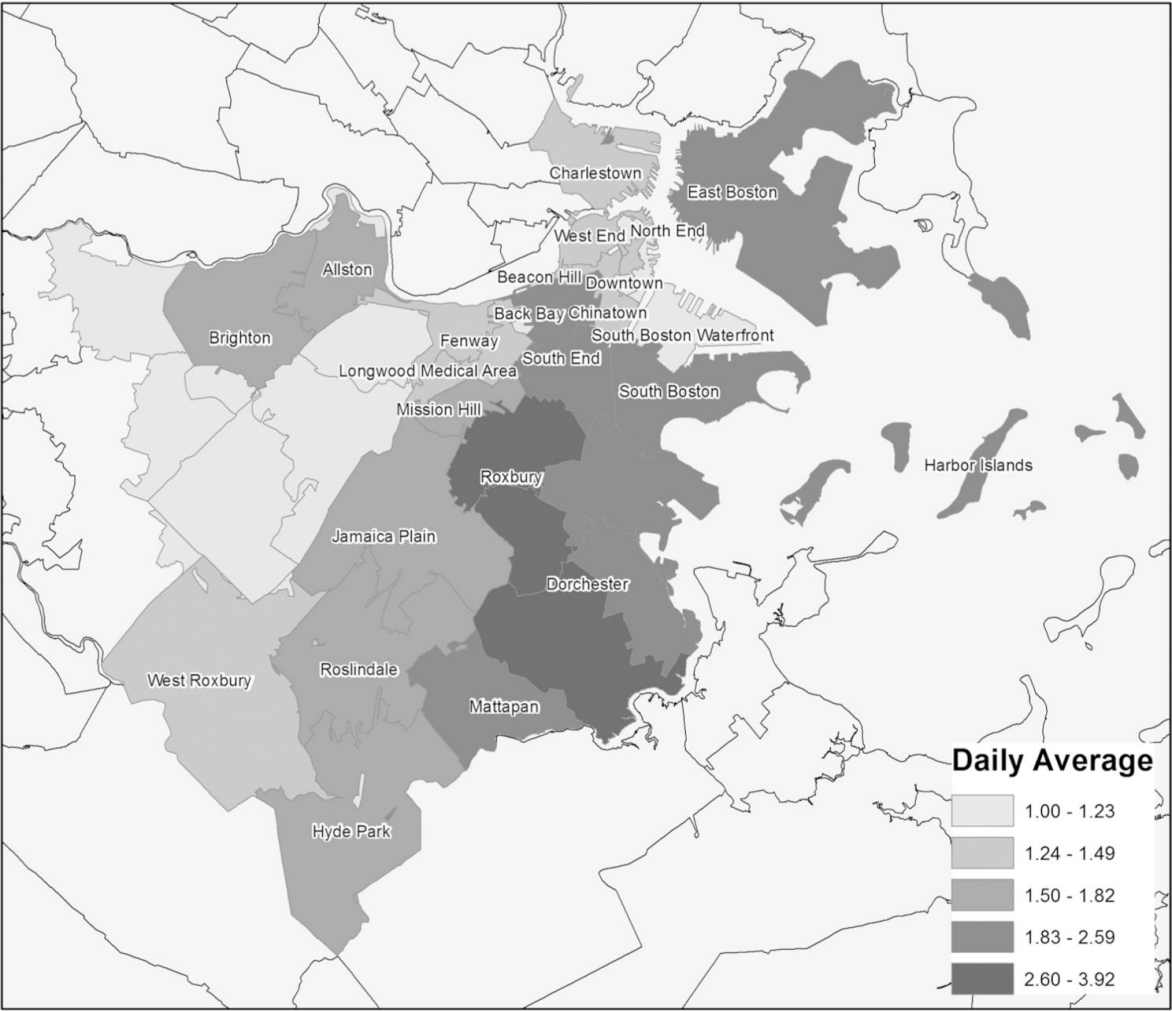
Mapping of the average daily violent crimes (between July 2012 and February 2017) per zip-code area in Boston

**Fig. 4:**
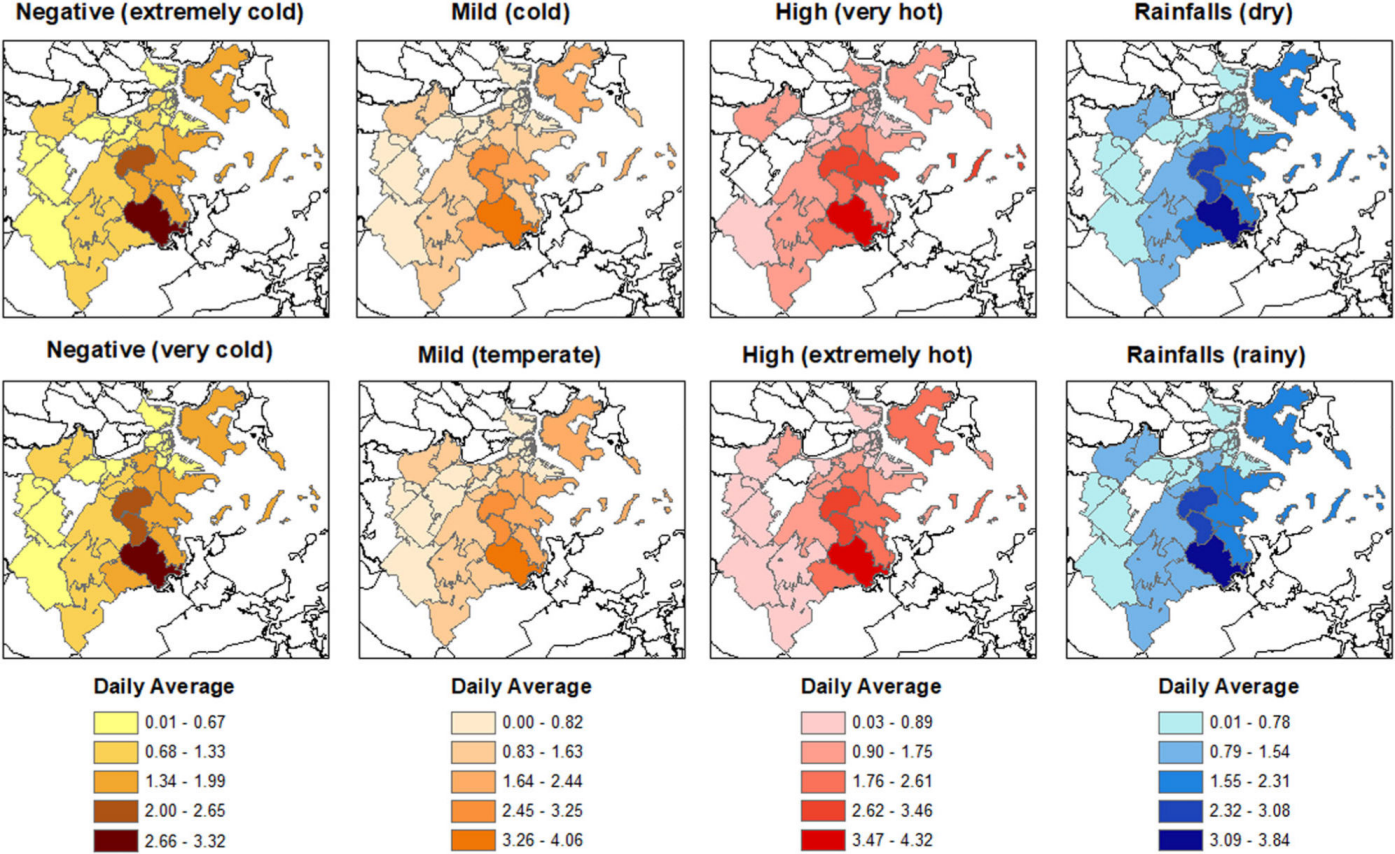
Spatial description: Mapping of the average daily violent crimes (between July 2012 and February 2017) per zip-code area for different exposure levels across the four hypothetical experiments

For each hypothetical experiment, we present estimated average exposure effects of different exposure levels on the total violent crime counts in Boston. However, there is spatial heterogeneity in crime counts by zip-code area (see Fig. 3). The mapping of the average daily violent crimes by zip-code area across hypothetical experiments (see Fig. 4) suggests that these variations are not homogeneous across experiments. When we compare the average daily violent crimes of days with low exposure level (upper panels) to days with a higher exposure level (lower panels), we observe for the Negative, Mild, High and Precipitation hypothetical experiments: (1) increases in Dorchester, (2) increases in Dorchester, South Boston, South End, and Fenway, (3) decreases in Roslindale, Hyde Park, Charlestown, West End, and Brighton, as well as increases in East Boston, Dorchester, and Longwood Medical area, and (4) decreases in Mattapan and Chinatown, respectively.

## Discussion

**Fig. 5:**
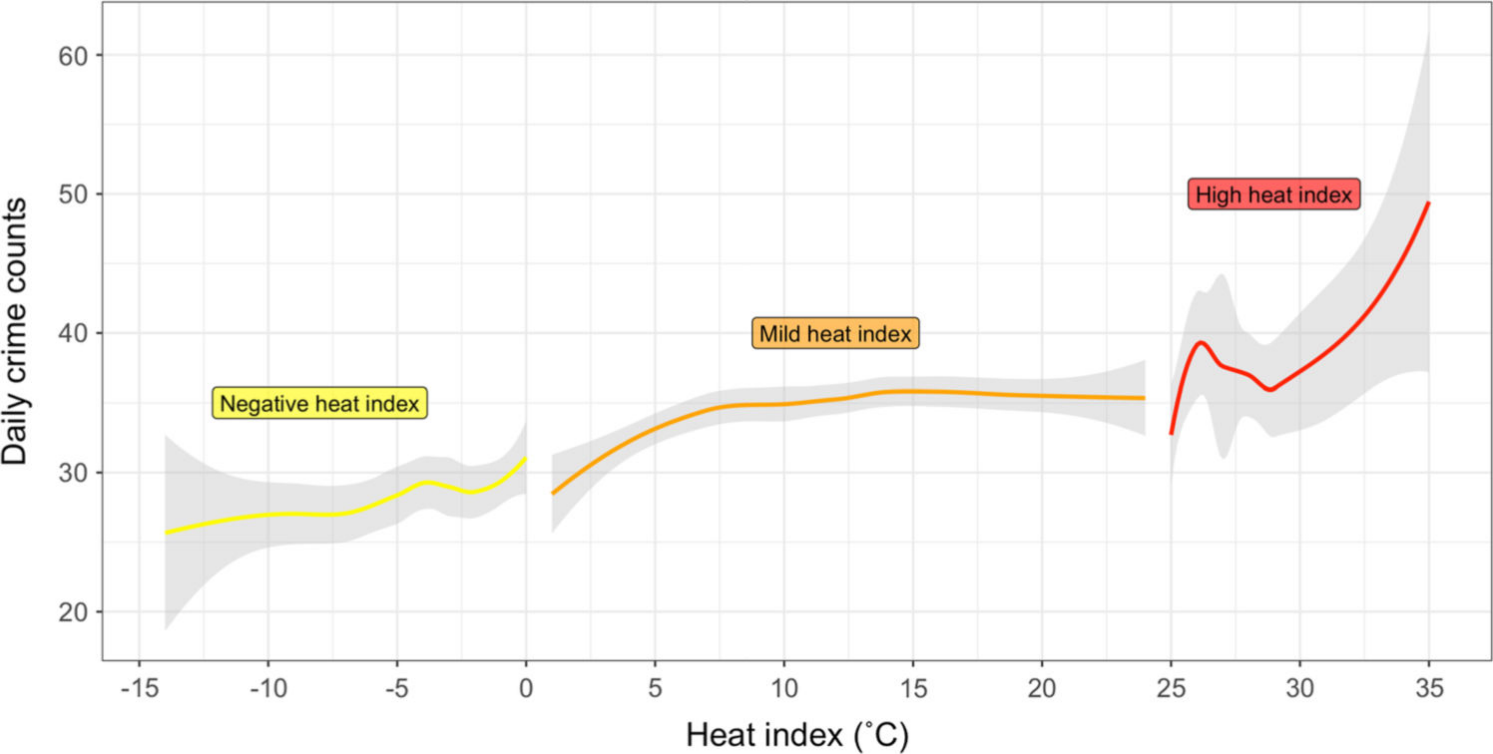
Graphical abstract: Smooth LOESS curves for the three hypothetical experiments (Negative, Mild, and High) focusing on the effects of different heat index exposure levels, fitted with the after propensity score matching samples

Our first research focus is to investigate the temperature–violent crime relationship in Boston. We observe a significant increase in daily violent crimes when moving from extremely cold days to temperate days. However, once the heat indexes are high, we do not observe any significant changes in trends in violent crimes frequency as the heat index exposure increases (see Fig. 5). Our second research focus was to examine whether rainfall decreased daily violent crime counts. Clearly, the occurrence of rainfall tends to decrease total daily violent crimes and aggravated assaults. To illustrate and make the effect sizes reported in our study more intuitive, we calculate an absolute change in crime count had thirty cold days been temperate in the Mild heat index experiment maintaining the covariates (e.g., population, length of daylight, or police deployment) the number of days in the other experiments constant. According to our results, if 30 cold days had been temperate, 56.4 (30 × 1.88) additional violent crimes would have occurred, in Boston on average.

The results on the effects of different heat exposures on larceny counts supports the routine activities theory. Indeed, not only aggressive behavior (e.g., violent crimes), but also other criminal activities such as property crime can be affected by varying heat exposures. Furthermore, the observed spatial heterogeneity in violent crime counts suggests that neighborhood differences should also be investigated. For example, the heat-neighborhood interaction could be precisely estimated by reconstructing hypothetical experiments with two conditions, (i.e., heat and neighborhood), each with two-levels (i.e., low/high heat, Downtown/Dorchester neighborhood). Matching techniques for the hypothetical reconstruction of multiple treatment experiments with observational data are discussed in Lopez and Gutman (2017).

It is important to note that interpretation should be restricted to days that remain in the sample after matching, these are the finite population of days between 2012 and 2017 with Bostonian weather characteristics (see Table S7). The data do not provide direct information for unmatched days. Cautiousness regarding extrapolation to days with covariate values beyond values observed in the matched data is necessary. In contrast to other studies interested in the effect of weather variations on criminal or aggressive behavior (Cohn and Rotton, 1997; Rotton and Cohn, 2000a; Cohn and Rotton, 2000, 2005; Schinasi and Hamra, 2017), this study does not provide any estimation of an exposure-response curve. This is a choice we made to be able to use the Rubin Causal Model with more transparent and plausible assumptions given our observational data. Environmental scientists interested in precise causal analysis and inference should not directly model observed data but instead consider a subset of their data exhibiting covariate balance, thereby approximating a hypothetical randomized experiment, in which valid causal effects can be estimated (Rubin, 2008). Generalizing causal effects is a complicated task. Directly modeling observed data from multiple cities can lead to misleading results because the socioeconomic, cultural, and climatic conditions can vary dramatically, and therefore cannot be guaranteed to be modeled correctly. We applied the same analysis strategy to Los Angeles (LA) crime data, a city with a smaller weather spectrum (see Supplementary Material G). The analysis of the weather–crime relationship in LA reveals the existence of a stronger heat–crime relationship than Boston. The results suggest that on average, 6.15 more (95% posterior interval: [3.74; 8.54]), violent crimes occur in LA during cold/temperate days (HI ≤ 17 °C) as compared to hotter days (HI > 17 °C). Interestingly, we observe no evidence for a rain–crime relationship. These divergent results show it is not straightforward to provide estimates generalizing the crime–weather relationship. To enhance crime prevention, the identification of causal factors for crimes should be conducted at the city, or even neighborhood, levels.

As opposed to Agnew (2011); Mares (2013a); Ranson (2014); Levy et al. (2017), our research question does not assess the direct impact of climate change on crimes, but proposes an approach to estimate the effect of weather variations on daily crime counts. Our causal inference approach can be extended to estimate the effects of anomalous weather patterns (as suggested by Mares (2013a)). We believe that this work is a contribution to the field of environmental health because crimes can have adverse effects on mental or physical health. Finding environmental explanations (e.g., weather condition variations), to crime outcomes will contribute to the public health debate on this issue. The results of this study affirm that when considering the economic, sociological, or medical impact of climate change; the effects of weather variations on criminal behavior should be taken into account.

The potential outcome framework coupled with a matchedsampling strategy we present was designed to understand the effect of plausible interventions. Therefore, we believe that interesting extensions to this study should consider not only a more precise spatial understanding of the weather–crime relationship in Boston, but also analyze whether location-specific police deployment with respect to specific weather conditions has an effect on violent crime counts.

Furthermore, although our approach does not suggest any direct intervention to reduce or stop crime incidence, we suggest interventions to alert the population and increase police deployment at certain locations when certain weather conditions can be predicted. Our study confirms that weather variables can have an effect on daily violent crime counts. Environmental factors should be included in crime prediction models to obtain more accurate criminality prevention. Some research focus on investigating the relationship between weather and mental health (Hansen et al., 2008). Other studies examine the effect of environmental exposures (e.g., air pollution) on the brain and behavior-related outcomes (e.g., criminal activity and unethical behavior) (Costa et al., 2017; Lu et al., 2018). Further research should be conducted on the relationships between weather and human behavior, as well as the interaction between temperature and air pollution on neurological outcomes based on observational and experimental data.

## Supplementary Material

Supplemental file

## Figures and Tables

**Table 1:** Literature summary on the crime-weather relationship: whereas routine activities theory helps explaining shifts in opportunity as weather changes, heat-aggression theories address individual motivators

	Routine activities	Heat-aggression

Experiments		

		- *Negative Affect Escape Model:* Baron and Bell (1976); Bell and Baron (1975) theorized from human experiments that aggression caused by negative affect (i.e., feelings of irritation, annoyance, or discomfort) increases with temperature increases until an unbearable temperature threshold is reached, after which people escape heat
		- *General (Affective) Aggression Model:* Anderson et al. (1995); Anderson and Bushman (2002) believe negative affect, aggressive thoughts, and physiological arousal are mediators to the effect of hot temperatures on aggressive tendencies
Observational studies		

Short-term effects	- Cohn and Rotton (1997, 2005) believe that, when time of the day is accounted for, while violence increases during pleasant weather, people are less likely to commit acts of violence when it is too hot (curvilinear relationship between temperature and aggression)	- Bushman et al. (2005) argue against Cohn and Rotton’s (1997; 2005) curvilinear relationship and for a linear relationship between temperature and assault, even during the hottest time of the day in hottest months of the year
	- *Social Escape and Avoidance Theory*: Rotton and Cohn (2000b) perceive pleasant weather as a promoter of social contact and inclement weather (especially heat and cold) as a factor reducing the chances of strangers coming into contact in public settings, instead inclement weather leads individuals to retreat to primary territories	
Long-term trends	- *Routine activities theory*: Cohen and Felson (1979) suggest crimes to occur when victims and offenders are brought into contact in the absence of controllers such as guardians, place managers, and intimate handlers such as parents, teachers, and employers	- Anderson et al. (1997) observed a positive relation between annual average temperature and serious and deadly assault, as well as between annual number of hot days and the size of usual Summer increase in violence
	- Rotton and Cohn (2003) found an association between annual temperatures and rates for assault, rape, robbery, burglary, and larceny	

## Footnotes

1 New Years Day, Martin Luther King Day, Washington’s Birthday, Good Friday, Memorial Day, Independence Day, Labor Day, Thanksgiving Day, Christmas

2 Boston Marathon, St. Patrick’s Day, Celtics’ Playoffs (basketball), Patriots’ Playoffs (superbowl)
