# Supplementary material for: Comparing apples to apples: an environmental criminology analysis of the effects of heat and rain on violent crimes in Boston
Source: Palgrave Commun. Author manuscript; Available in PMC 2019 Jul 29. (PMC6663090; doi:10.1057/s41599-018-0188-3)
Supplement: Supplemental file [file NIHMS1007774-supplement-Supplemental_file.pdf]

Supplementary Material: An environmental criminology analysis of the effects of heat and rain on violent crimes in Boston

-

-

A Data description

	Crimes (day count)	Heat Index (°C)	Precipitation (mm)	Windspeed (mph)
Fall	34.51<br>(7.93)	12.24<br>(6.7)	0.10<br>(0.28)	9.64<br>(3.24)
Winter	30.33<br>(7.67)	0.15<br>(5.4)	0.13<br>(0.27)	9.41<br>(3.61)
Spring	33.69<br>(7.65)	7.84<br>(6.6)	0.09<br>(0.22)	7.88<br>(3.09)
Summer	37.48<br>(7.45)	22.31<br>(3.9)	0.11<br>(0.33)	8.43<br>(2.17)

Table S1: Mean (standard deviation) daily counts of violent crimes and weather factors across seasons in Boston, MA, between July 2012 and February 2017

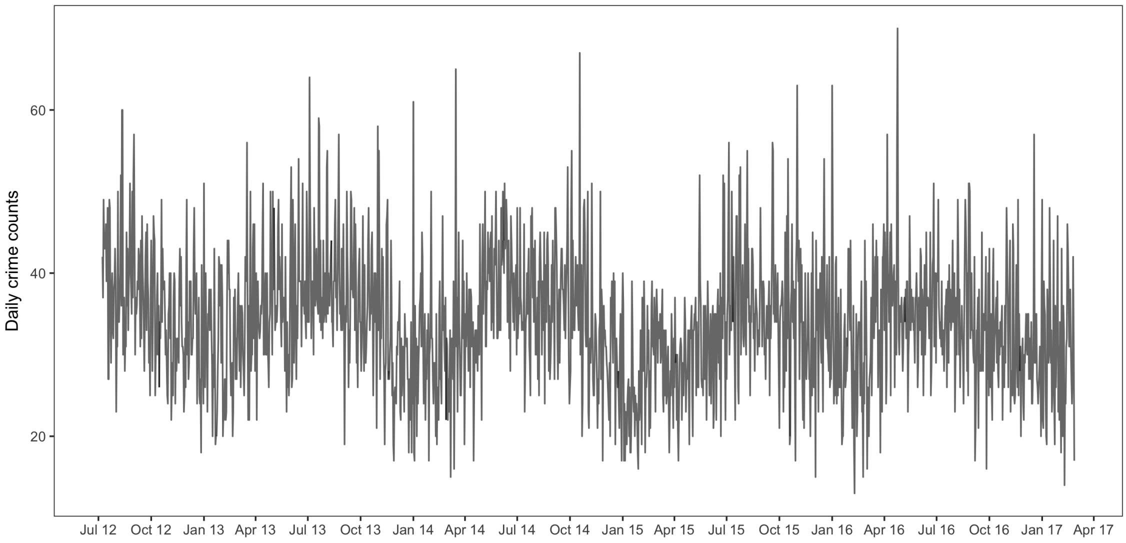

Figure S1: Daily reported violent crimes counts in Boston, MA, between July 2012 and February 2017

B Data segmentation for the four hypothetical experiments

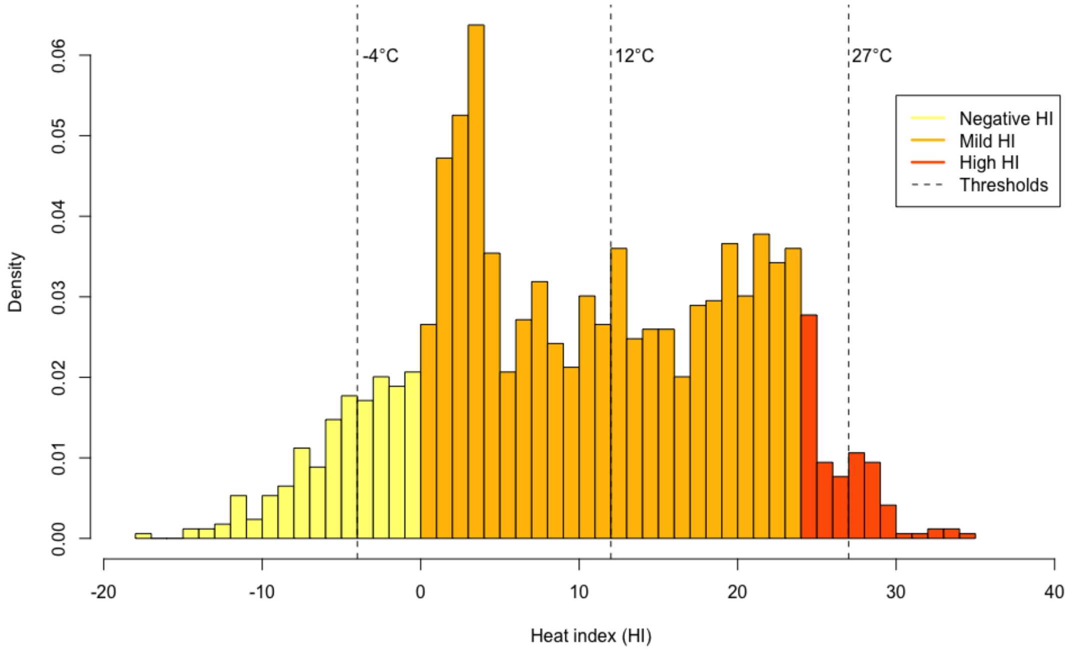

(a) Heat index distribution

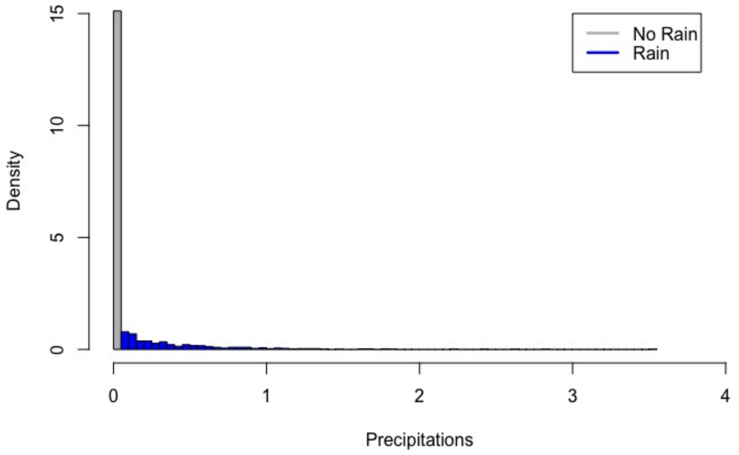

(b) Precipitations distribution

Figure S2: Exposure distributions and exposure levels segmentation for the hypothetical experiments

	Negative HI		Mild HI		High HI		Rainfalls	
Exposure (W_i)	0	1	0	1	0	1	0	1
Thresholds (T_i)	$\leq -4°C$	$> -4°C$	$\leq 12°C$	$> 12°C$	$\leq 27°C$	$> 27°C$	0	> 0
Nb. of days i	128	130	687	624	76	50	1027	668

Table S2: Segmentation of the units by exposure level for the four hypothetical experiments

	unit i	Date	Exposure (Z_i)	Treatment (W_i)	Y_i^{obs}	$Y_i(0)$	$Y_i(1)$
• Negative HI	1	2012.12.01	$-1°C$	1	41	?	41
	...	...	...	...	...	...	...
	260	2017-02-26	$-1°C$	1	31	?	31
• Mild HI	1	2012.07.09	24°C	1	37	?	37
	...	...	...	...	...	...	...
	1310	2017.02.17	5°C	0	17	17	?
• High HI	1	2012.07.08	27°C	0	42	42	?
	...	...	...	...	...	...	...
	124	2016.09.09	28°C	1	33	?	33
• Rainfalls	1	2012.07.08	0 mm	0	42	42	?
	...	...	...	...	...	...	...
	1695	2017.02.26	0.107 mm	1	17	?	17

Table S3: Potential outcome formulation with science table examples for the four hypothetical experiments

C Results

	**Number of units**	**Estimate of the AEE**	**95% interval**
• Negative Heat Index	190	**1.75**	**[0.34 ; 3.17]**
• Mild Heat Index	600	**1.88**	**[1.10 ; 2.66]**
• High Heat Index	74	2.19	[-0.36 ; 4.77]
• Rainfalls	1174	**-1.37**	**[-1.94 ; -0.79]**

Table S4: Primary results: Estimates of the average exposure effect (AEE) of different exposure levels on violent crimes across the four hypothetical experiments after multiply imputing the missing potential outcomes 10,000 times

Type of crime	**Hypothetical experiment**	**Number of units**	**Estimate of the AEE**	**95% interval**
Aggravated assault	• Negative HI	190	**0.91**	**[0.45 ; 1.38]**
	• Mild HI	600	**0.58**	**[0.30 ; 0.86]**
	• High HI	74	**1.31**	**[0.18 ; 2.41]**
	• Rainfalls	1174	**-0.35**	**[-0.55 ; -0.14]**
Larceny	• Negative HI	190	**1.59**	**[0.01; 3.09]**
	• Mild HI	600	**1.46**	**[0.65 ; 2.26]**
	• High HI	74	1.69	[-0.99 ; 4.43]
	• Rainfalls	1174	-0.54	[-1.15 ; 0.08]

Table S5: Exploratory results: Estimates of the average exposure effect (AEE) of different exposure levels on aggravated assaults and larceny across the four hypothetical experiments after multiply imputing the missing potential outcomes 10,000 times

D Overlap in covariate distributions

	• Negative HI		• Mild HI		• High HI		• Rainfalls	
Exposure (W_i)	0	1	0	1	0	1	0	1
PS range Before	[0.27 ; 0.88]	[0.25 ; 0.95]	[1.26×10^{-9} ; 0.99]	[0.01 ; 0.99]	[0.23; 0.70]	[0.23 ; 0.71]	[0.17 ; 0.88]	[0.19 ; 0.94]
PS range After	[0.32 ; 0.77]	[0.32 ; 0.77]	[0.02 ; 0.99]	[0.02 ; 0.99]	- -	- -	[0.19 ; 0.87]	[0.19 ; 0.86]
# deleted	3	13	91	13	-	-	3	10

Table S6: Propensity score (PS) range for the control and exposed days before and after outlying days deletion across the four hypothetical randomized experiments

E Balance in covariate distributions

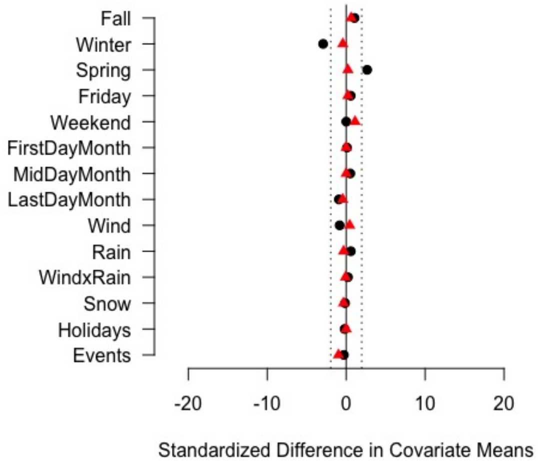

(a) Hypothetical Experiment 1: Negative heat index days

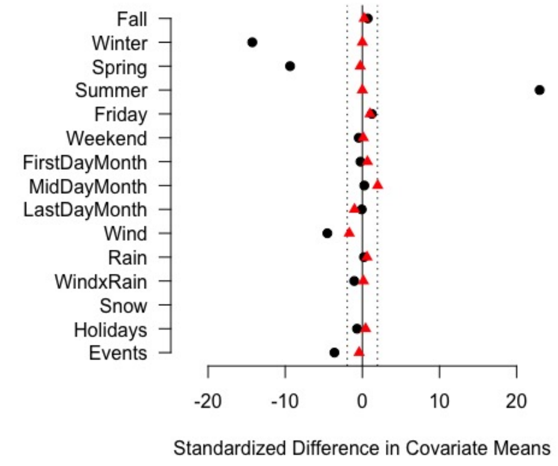

(b) Hypothetical Experiment 2: Mild heat index days

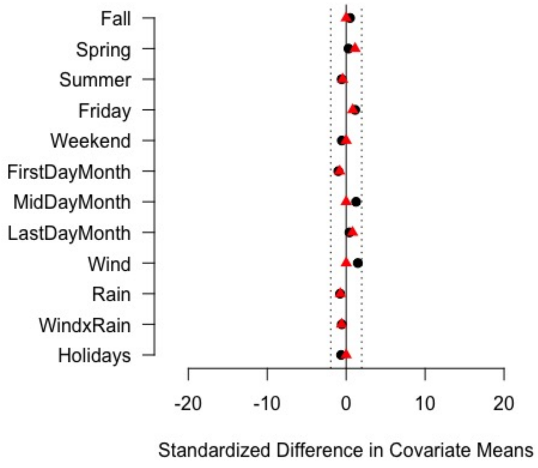

(c) Hypothetical Experiment 3: High heat index days

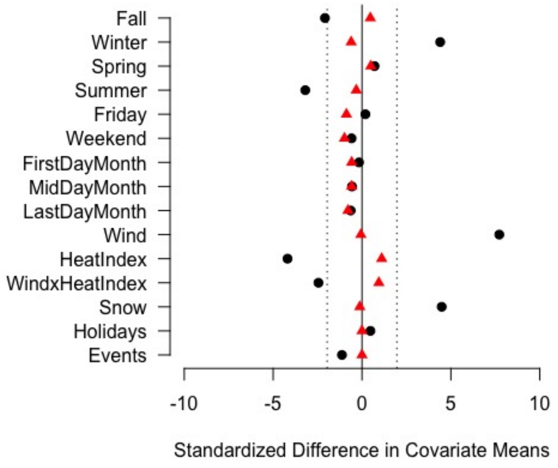

(d) Hypothetical Experiment 4: Rainfalls

Figure S3: "Love" plots: Background covariates standardized mean difference for the before (•) and after (▲) propensity score matching with caliper 0.1 across the four hypothetical randomized experiments

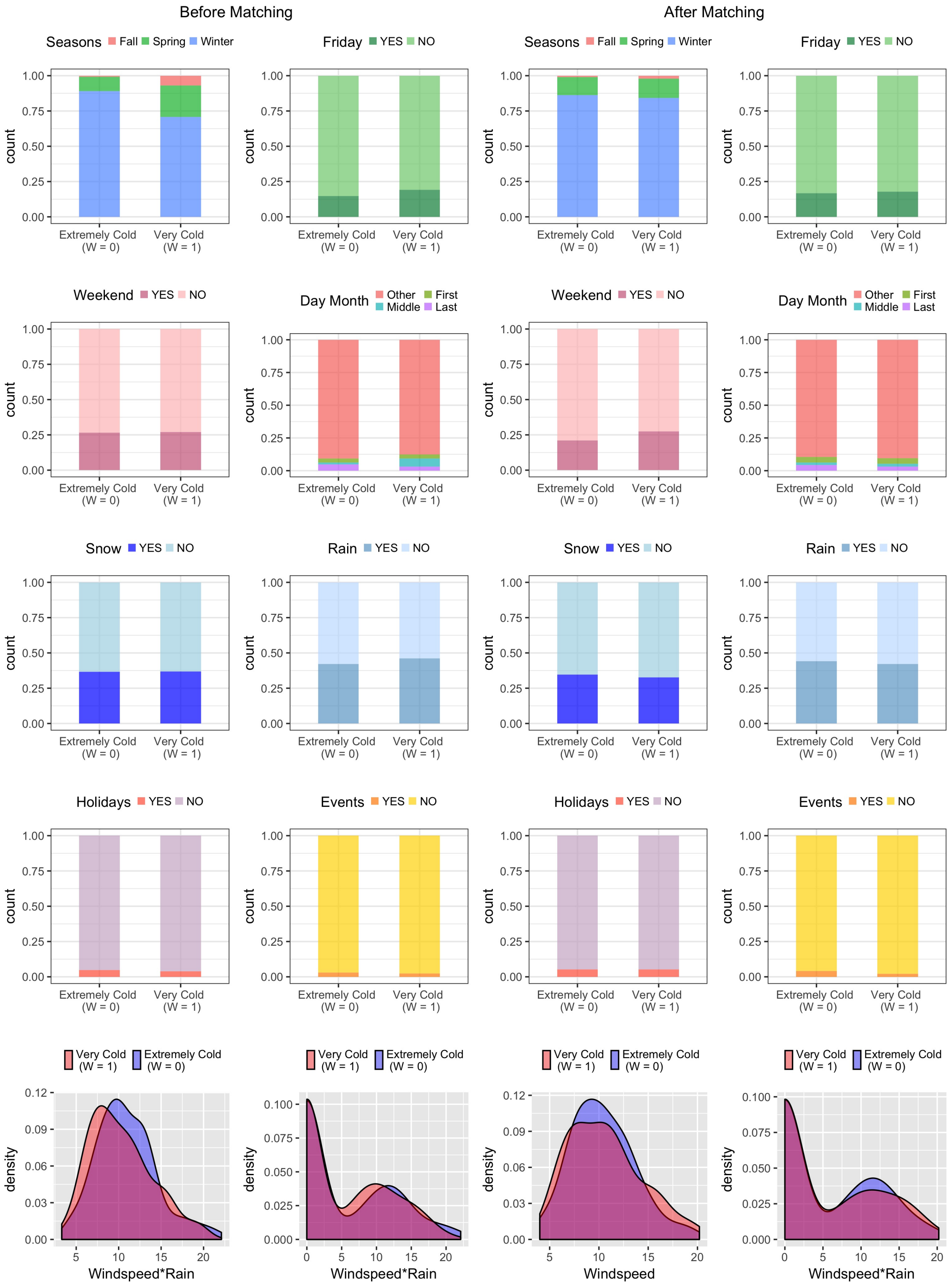

Figure S4: Negative Heat Index days experiment empirical distributions of the background covariates among control and exposed days in the original dataset (left panels) and after propensity score matching (right panels)

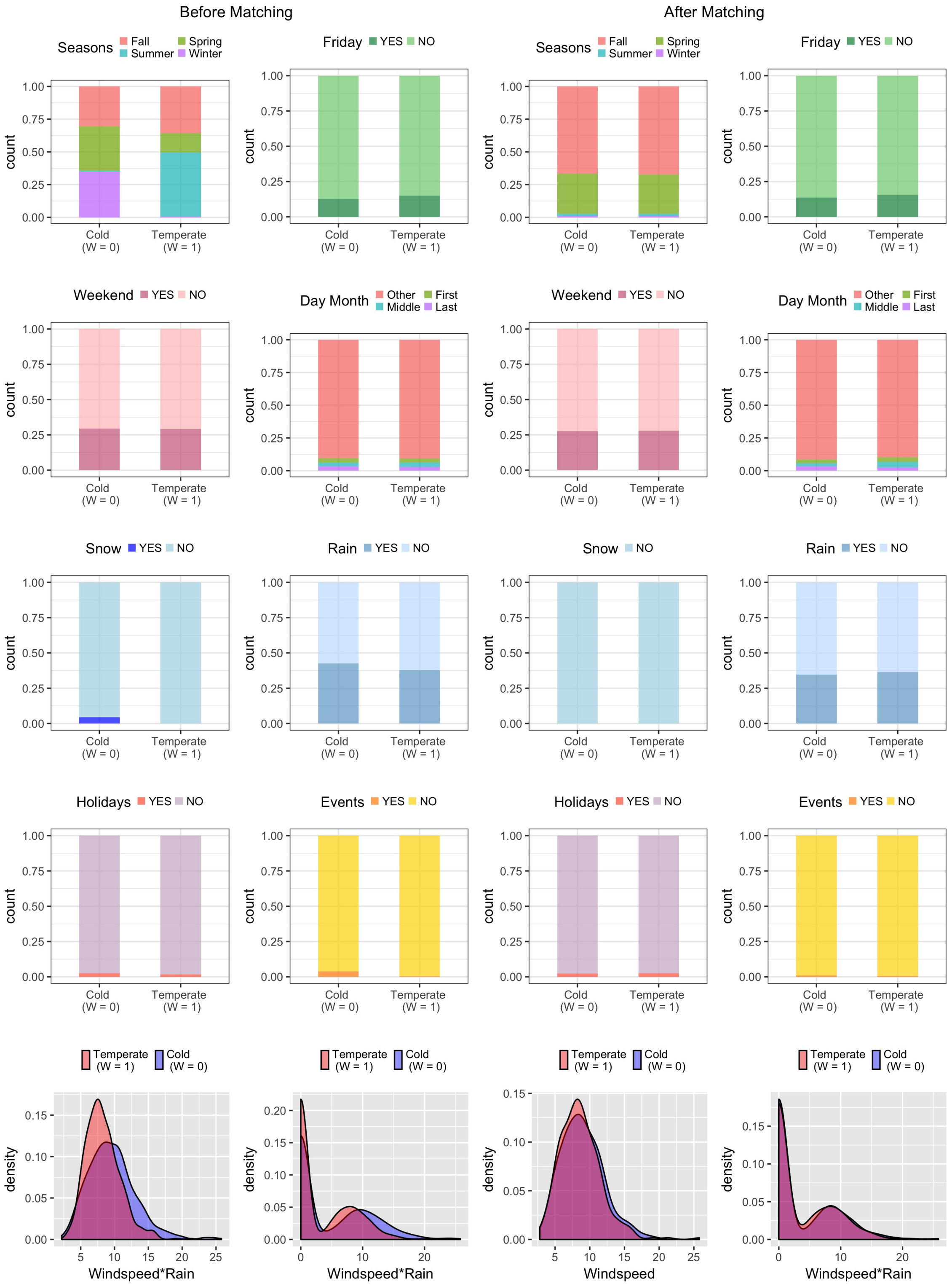

Figure S5: Mild Heat Index days experiment empirical distributions of the background covariates among control and exposed days in the original dataset (left panels) and after propensity score matching (right panels)

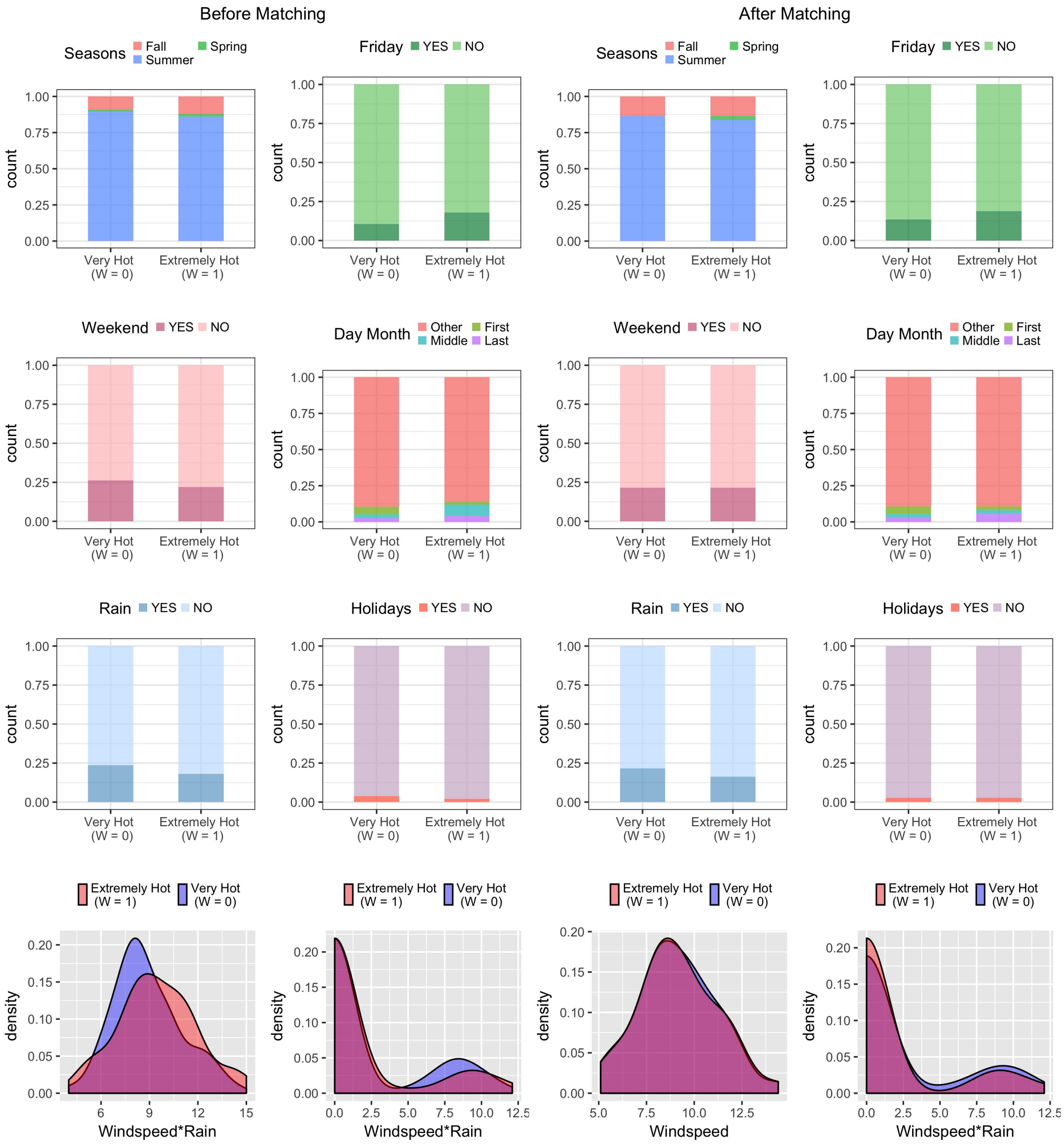

Figure S6: High Heat Index days experiment empirical distributions of the background covariates among control and exposed days in the original dataset (left panels) and after propensity score matching (right panels)

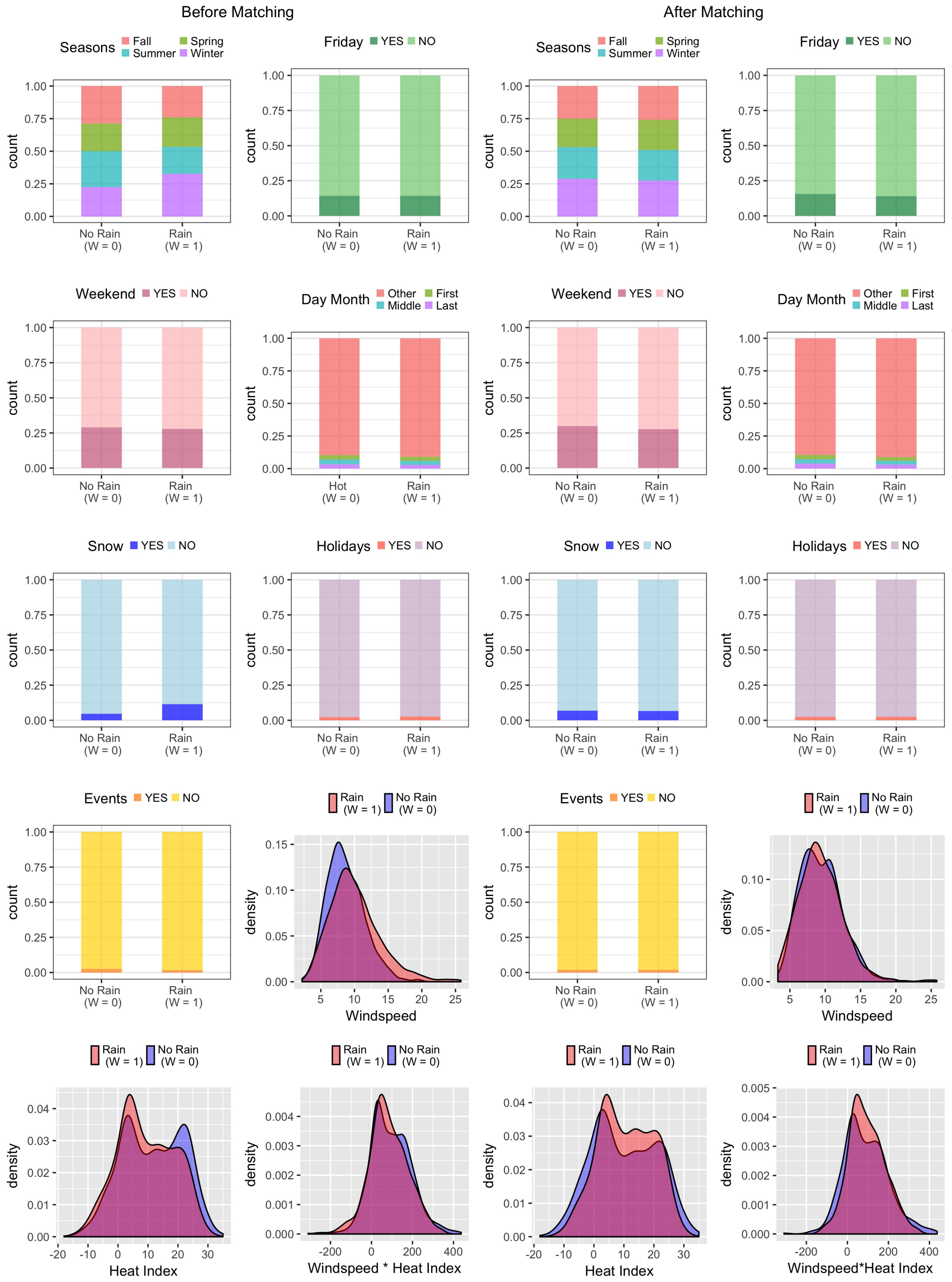

Figure S7: Rainfalls experiment empirical distributions of the background covariates among control and exposed days in the original dataset (left panels) and after propensity score matching (right panels)

	Hypothetical experiment 1				Hypothetical experiment 2				Hypothetical experiment 3				Hypothetical experiment 4			
	Before Matching		After Matching		Before Matching		After Matching		Before Matching		After Matching		Before Matching		After Matching	
	Controls Mean n = 128	Exposed Mean n = 130	Controls Mean n = 95	Exposed Mean n = 95	Controls Mean n = 687	Exposed Mean n = 624	Controls Mean n = 300	Exposed Mean n = 300	Controls Mean n = 76	Exposed Mean n = 50	Controls Mean n = 37	Exposed Mean n = 37	Controls Mean n = 1027	Exposed Mean n = 668	Controls Mean n = 587	Exposed Mean n = 587
**Weather variables**																
Windspeed; mean	10.97	10.29	10.42	10.61	9.60	8.32	8.94	8.65	8.85	9.47	9.16	9.17	8.71	10.08	9.39	9.38
Heat Index; mean	-	-	-	-	-	-	-	-	-	-	-	-	11.50	9.40	10.19	10.72
Rain	0.42	0.46	0.44	0.42	0.43	0.38	0.35	0.36	0.24	0.18	0.22	0.16	-	-	-	-
Snow	0.37	0.37	0.35	0.33	0.04	0.00	0.00	0.00	-	-	-	-	0.05	0.12	0.07	0.07
**Seasons**																
Fall	0.01	0.07	0.01	0.02	0.30	0.36	0.67	0.67	0.13	0.13	0.14	0.14	0.29	0.24	0.25	0.26
Winter	0.89	0.70	0.86	0.84	0.35	0.01	0.01	0.01	0.00	0.00	0.00	0.00	0.22	0.33	0.28	0.27
Spring	0.10	0.22	0.13	0.14	0.34	0.15	0.31	0.30	0.01	0.02	0.00	0.02	0.21	0.23	0.23	0.22
Summer	0.00	0.00	0.00	0.00	0.01	0.49	0.02	0.02	0.90	0.85	0.86	0.84	0.28	0.21	0.24	0.24
**Day of Week**																
Friday	0.15	0.19	0.17	0.18	0.13	0.15	0.14	0.16	0.11	0.19	0.14	0.19	0.14	0.14	0.16	0.14
Weekend	0.26	0.27	0.21	0.27	0.30	0.29	0.28	0.28	0.25	0.23	0.22	0.22	0.29	0.28	0.30	0.28
**Day of Month**																
FirstDayMonth	0.03	0.03	0.04	0.04	0.03	0.03	0.03	0.04	0.05	0.02	0.05	0.03	0.03	0.03	0.03	0.03
MidDayMonth	0.02	0.06	0.02	0.02	0.03	0.03	0.02	0.04	0.03	0.08	0.03	0.03	0.04	0.04	0.03	0.03
LastDayMonth	0.05	0.03	0.04	0.03	0.03	0.03	0.03	0.02	0.03	0.04	0.03	0.05	0.03	0.03	0.04	0.03
**Additional variables**																
Holidays	0.05	0.04	0.05	0.05	0.02	0.02	0.02	0.03	0.04	0.02	0.03	0.03	0.02	0.03	0.03	0.03
Events	0.03	0.02	0.04	0.02	0.04	0.01	0.01	0.01	0.00	0.00	0.00	0.00	0.03	0.02	0.02	0.02

Table S7: Background covariates balance before and after matching (all the means are proportions unless noted)

F Missing potential outcome models for the exploratory results

F.1 Aggravated assault

$Y_i^{obs} \sim NB(\mu_i, \phi)$

$$\mu_i = exp(\eta_i) \qquad and \qquad \eta_i = \boldsymbol{\beta^T} \ \mathbf{X_i^{HExp_k}} \qquad \text{for k = 1, ..., 4}$$

$$\mathbf{X_i^{HExp_1}} = (1, FirstDayMonth_i, MidDayMonth_i)$$
$$\mathbf{X_i^{HExp_2}} = (1, Weekend_i, Rain_i, Wind_i * Rain_i)$$
$$\mathbf{X_i^{HExp_3}} = (1, Weekend_i)$$
$$\mathbf{X_i^{HExp_4}} = (1, Friday_i, Weekend_i, Winter_i, HeatIndex_i)$$

Priors:
$\phi \sim$ *Half-Cauchy*$(0,\ 5)$ $\qquad \beta_0 \sim N(0, 5) \qquad \beta_{1,\dots,4} \sim N(0, 2.5)$

F.2 Larceny

$Y_i^{obs} \sim NB(\mu_i, \phi)$

$$\mu_i = exp(\eta_i) \qquad and \qquad \eta_i = \boldsymbol{\beta^T} \ \mathbf{X_i^{HExp_k}} \qquad \text{for k = 1, ..., 4}$$

$$\mathbf{X_i^{HExp_1}} = (1, Friday_i, Snow_i)$$
$$\mathbf{X_i^{HExp_2}} = (1, Fall_i, Friday_i, Holidays_i, Wind_i * Rain_i)$$
$$\mathbf{X_i^{HExp_3}} = (1, Weekend_i)$$
$$\mathbf{X_i^{HExp_4}} = (1, Friday_i, Weekend_i)$$

Priors:
$\phi \sim$ *Half-Cauchy*$(0,\ 5)$ $\qquad \beta_0 \sim N(0, 5) \qquad \beta_{1,\dots,4} \sim N(0, 2.5)$

G Los Angeles Analysis

Weather conditions given the monitoring station located at Los Angeles International airport during study period were obtained from the Climate Data Online provided by the National Oceanic and Atmospheric Administration of the U.S. Department of Commerce (NOAA, data available at https://www.ncdc.noaa.gov/cdo-web). The crime data come from crime incident reports (between January 2010 and March 2018) collected by the Los Angeles Police Department and made available on the City of Los Angeles Data Portal (data available at https://data.lacity.org). Details on the location and time of day are given for all reported crimes.

The background covariates included in the two hypothetical experiments are the binary variables: *Fall*, *Winter*, *Spring*, *Summer*, *Friday*, *Weekend*, *FirstDayMonth* (i.e., first day of the month), *MidDayMonth* (i.e., 15st day of the month), *LastDayMonth* (i.e., last day of the month), *Holidays*[1], and *Events*[2] as well as the continuous variable *Wind* (i.e., windspeed). In the first experiments, concentrating on the effects of heat, *Rain* (i.e., rainfalls occurrence) is an additional binary background covariate. In the last experiment, concentrating on the effect of rainfalls, *HeatIndex* plays the role of a background covariate. Notice that as compared to the Boston analysis, no *Snow* covariate is included because it does not snow in Los Angeles.

[1] New Years Day, Martin Luther King Day, Washington's Birthday, Good Friday, Memorial Day, Independence Day, Labor Day, Thanksgiving Day, Christmas

[2] Los Angeles Marathon, Lakers' Playoffs (basketball), Clippers' Playoffs (basketball)

G.1 Data segmentation for the two hypothetical experiments

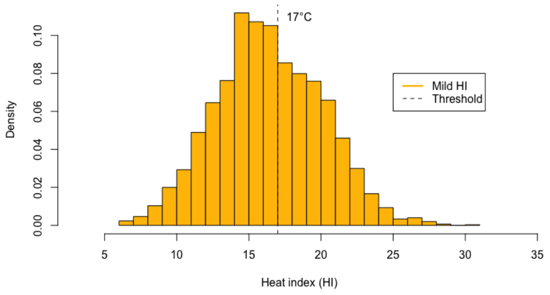

(a) Heat index distribution

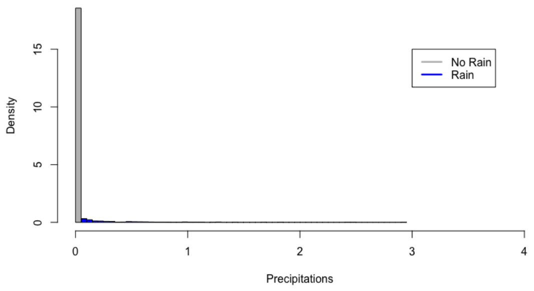

(b) Precipitations distribution

Figure S8: Exposure distributions and exposure levels segmentation for the hypothetical experiments

	• Mild HI		• Rainfalls	
Exposure (W_i)	0	1	0	1
Thresholds (T_i)	$\leq 17°C$	$> 17°C$	0	> 0
Nb. of days i	1743	1260	2680	323

Table S8: Segmentation of the units by exposure level for the two hypothetical experiments

G.2 Results

	**Number of units**	**Estimate of the AEE**	**95% interval**
• Mild Heat Index	972	**6.15**	**[3.74 ; 8.54]**
• Rainfalls	572	-2.23	[-5.88 ; 1.48]

Table S9: Primary results: Estimates of the average exposure effect (AEE) of different exposure levels on violent crimes across the two hypothetical experiments after multiply imputing the missing potential outcomes 10,000 times

G.3 Overlap in covariate distributions

	• Mild HI		• Rainfalls	
Exposure (W_i)	0	1	0	1
PS range Before	[0.11 ; 0.86]	[0.06 ; 0.86]	[0.001 ; 0.94]	[0.003 ; 0.98]
PS range After	[0.07 ; 0.86]	[0.07 ; 0.86]	[0.003 ; 0.92]	[0.003 ; 0.91]
# deleted	246	0	25	9

Table S10: Propensity score (PS) range for the control and exposed days before and after outlying days deletion across the two hypothetical randomized experiments

G.4 Balance in covariate distributions

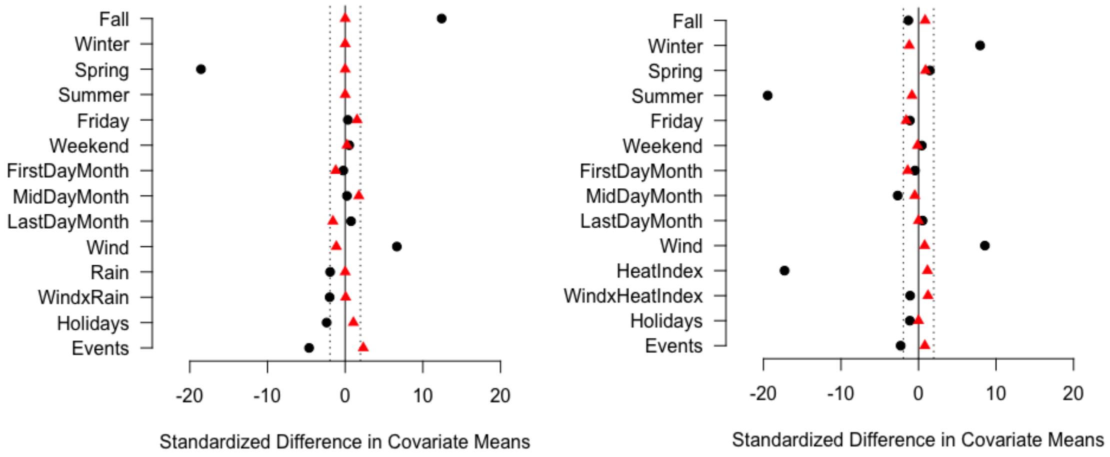

(a) Hypothetical Experiment 1: Mild heat index days

(b) Hypothetical Experiment 2: Rainfalls

Figure S9: "Love" plots: Background covariates standardized mean difference for the before (●) and after (▲) propensity score matching with caliper 0.1 across the two hypothetical randomized experiments

G.5 Missing potential outcomes models

$Y_i^{obs} \sim NB(\mu_i, \phi)$

$$\mu_i = exp(\eta_i) \qquad and \qquad \eta_i = \boldsymbol{\beta^T} \; \mathbf{X_i^{HExp_k}} \qquad \text{for k} = 1, 2$$

$$\mathbf{X_i^{HExp_1}} = (1, Winter_i, Fall_i, Friday_i, Weekend_i, FirstDayMonth_i, Wind_i, Holidays_i, Events_i)$$
$$\mathbf{X_i^{HExp_2}} = (1, Fall_i, Friday_i, Weekend_i, FirstDayMonth_i, MidDayMonth_i, Wind_i, WindxHeatIndex_i, Holidays_i)$$

Priors:

$\phi \sim \textit{Half-Cauchy}(0,\ 5) \qquad \beta_0 \sim N(0, 5) \qquad \beta_{1,\ldots,8} \sim N(0, 2.5)$
